# Resolution Limits of Analyzers and Oscillatory Systems

**DOI:** 10.6028/jres.067A.048

**Published:** 1963-10-01

**Authors:** Edith L. R. Corliss

## Abstract

This paper considers the resolution limits of those analyzers and oscillatory systems whose performance may be represented by a second-order differential equation. The “signal uncertainty” product Δ*f*·Δ*t* is shown to be controlled by the ability of a system to indicate changes in energy content. The discussion refers the functioning of the system to a signal space whose coordinates are energy, frequency, and time. In this signal space, the product of the resolution limits, *U *= (Δ*E*/*E*_0_) (Δ*f*/*f*_0_) (Δ*t*/*T*_0_) is the volume of a region within which no change of state in the system may be observed. Whereas the area element Δ*f*·Δ*t* is freely deformable, no operations upon either Δ*f* or Δ*t* can further the reduction of the energy resolution limit. Thus *U* is irreducibly fixed by the limiting value of Δ*E*/*E*_0_. By considering the effects of noise upon Δ*E*/*E*_0_, and thus upon *U*, the paper demonstrates the rise of statistical features as signal-to-noise ratios decrease.

Functional relationships derived from Δ*E*/*E*_0_ and *U* are tabulated. These equations facilitate computation of the limits of observable changes of state in a system, and they provide guidance for the design of experiments to apportion the uncertainties of measurement of transient phenomena as advantageously as possible. A reference bibliography and appendices giving somewhat detailed proofs are included.

The basis of this paper is the consideration that the indication of most instruments used in measurement represents either the storage of energy or the flow of power. The least changes that the instruments can indicate, therefore, are controlled by the smallest discernible change in energy storage or power flow.

The subject of the resolution limits of measuring instruments in terms of the least amounts of frequency change and the least time interval in which a change may be detected have been treated by several authors, among whom one may cite as examples Gabor, Kharkevich, and Brillouin—and, while this paper was being revised, Pimonow.^1^ The present author has also discussed this relationship for scanning analyzers, and indicated that there were circumstances in which limitations were introduced by the presence of a least discernible increment of power or energy.^2^ These papers (ref. 2) are quoted, in addition to the prior work, by Pimonow.

Gabor pointed out, by analogy to quantum theory, that there was a “quantum” of information that could be described by the product of differentials representing the least discriminable increment of frequency that could be observed in an increment of time. This relation arose from the application of the Fourier transform to relate an increment of time to its corresponding increment in the frequency domain. The product

Δf⋅Δt≈1

was defined by Gabor as the “Logon.” The fact that, as he says, the product is “of the order of unity” is a consequence of the particular normalization he used in computing the Fourier transform for Gaussian pulses. A similar relation was presented by Brillouin, but as he computed Δ*t* in terms of the half-powers of brief, symmetrical pulses, he found a somewhat different normalization factor, and obtained

$$\Delta f\cdot \Delta t=\frac{1}{4\pi }.$$

Kharkevich adopted a somewhat more general expression for this equation, also in terms of a normalized Fourier transform, by first writing

Δf⋅Δt=A

and computing *A* for pulses of various forms. He remarked that *A* might differ if other criteria were chosen, but related *A* only to the form of the signal. Further, he pointed out that *A* is independent of the, damping of the system.

The studies by Gabor. Kharkevich, and Brillouin were all carried out for essentially noiseless systems. This paper, on the other hand, does not normalize for unit energy, but considers the energy storage and dissipation in systems whose performance may be described by a linear differential equation of the second order. Thus, by dealing with the energy stored as well as with the time and frequency we are able to study the response of a system to signals other than variously shaped pulses of unit energy, and to signals in noise as well as to noiseless systems.

We can also consider, in this way, the case which has been omitted from the previous work: the response of the system which may in itself have a “least count”^3^ or inherent internal noise.

We introduce the means for taking into account the presence of noise with a signal, internal noise in an analyzer, or the least discernible indication of the analyzer (which may be a step limitation—such as a digital step, or a reading limit) by discussing the limits of the analyzer’s performance as being fixed by the least change in energy storage, Δ*E*, that can be resolved under the circumstances of analysis. Several conditons may combine to fix the value of Δ*E*. For example, an analyzer having appreciable self-noise may be used to detect a signal in noise. As a rule, the internal and external noise sources in that case would be incoherent, and the sum of the noise energies stored in the analyzer from those sources would fix the value of Δ*E*.

The system for which this discussion is carried out is a system whose working may be described by a linear differential equation of the second order. This behavior is common to many physical systems occurring in nature, and to many instruments used to observe natural phenomena. All of these systems share the same properties, because they are properties inherent in the differential equation that describes them. By virtue of the second-order term, they may be seen to be capable of storing oscillatory energy reversibly. They will respond with a sinusoidal output after excitation by shock or noise. Under sinusoidal excitation, they will respond selectively to excitation of various frequencies.

Systems to be discussed in this paper are those in which the storage of energy occurs in the coordinates describing the system: these systems are described by a second-order differential equation with constant coefficients; i.e., the system parameters are not affected by the energy storage process. By invoking the Boltzmann-Ehrenfest adiabatic principle,^4^ it is also possible to apply many of these equations to systems in which energy is stored by a change of parameters. However, this matter has not been investigated in detail.

Although most of the systems to which the second-order differential equation is applicable take part in time-varying phenomena, there is nothing inherent that restricts the equation to functions of time. Certain spatial distributions also may be described by the equation—such as the magnetization on magnetic tape, some types of optical images, and some diffraction effects. Thus, although this paper will deal with application of the equation to time-varying phenomena, its conclusions are also applicable, with a judicious choice of variables, to spatial distributions.

For the sake of a coherent structure upon which to base this paper, we choose a mechanical system of inertia, *M*, dissipation (proportional to velocity) *D*, and coefficient of restitution, *k*. This system has a single degree of freedom, along the coordinate *x*, and its force-free behavior is given by solutions of the homogeneous equation:

$$M\ddot{x}+D\ddot{x}+kx=0.$$

When energy is stored in the system, it is dissipated at a mean rate which bears a constant relationship to the amount of energy stored. This constant is a function of the parameters of the system. In terms of dissipation, the constant is frequently expressed by the relative damping, *γ*, the ratio of the damping of the system to the critical damping for no oscillation. A reciprocal quantity, the “figure of merit,” *Q*, is commonly used in communication problems. These two constants are related through the equation:

$$\gamma =\frac{1}{2Q}.$$

Because we are more concerned here with storage than with dissipation, the quantity *Q* will be used in the discussion.

The conventional definition for *Q* applies when the system is driven at its resonance frequency; at other frequencies the ratio of the storage of energy to the rate of dissipation depends upon the driving frequency. When the system is free of excitation, the conventional definition of *Q* again applies. This value of *Q* will be denoted as *Q*_0_, to distinguish it from the more general definition of *Q* to be applied in the appendix. Defining as the natural frequency of the system the quantity *f*_0_, which is the natural frequency of the system in the absence of damping,

$$f_{0}=\frac{1}{2\pi }\sqrt{\frac{k}{M}}$$

and

$$Q_{0}=\frac{\text{Peak}\;\text{energy}\;\text{stored}\;\text{at}\;\text{the}\;\text{natural}\;\text{frequency}}{\text{Energy}\;\text{dissipated}\;\text{per}\;\text{cycle}\;\text{at}\;\text{the}\;\text{natural}\;\text{period}}$$

In terms of the parameters of the system, this definition of *Q*_0_ is equivalent to the ratio:

$$Q_{0}=\frac{2\pi f_{0}M}{D}=\frac{k}{2\pi f_{0}D}=\frac{\sqrt{kM}}{D}.$$

The differential equations for the transient and driven response of the system can be expressed in terms of *Q*_0_, *D*, and *f*_0_. For the force-free equation:

$$\frac{\ddot{x}}{2\pi f_{0}}+\frac{\dot{x}}{Q_{0}}+2\pi f_{0}x=0$$

and, for the driven response of the system to a sinusoidal force of amplitude *A* and frequency *f*:

$$\frac{\ddot{x}}{2\pi f_{0}}+\frac{\dot{x}}{Q_{0}}+2\pi f_{0}x=\frac{A}{Q_{0}D}e^{j2\pi ft}.$$

Letting the ratio of the driving frequency to the natural undamped frequency be represented by

$$\phi =f/f_{0}$$

we can write down the phase relation between the driving force and the resulting motion. The phase angle, *θ*, when the steady-state condition has been attained is given by

$$\theta =\tan^{-1}\frac{\phi }{Q_{0}\left(1-\phi^{2}\right)}$$

and the energy stored in the steady-state condition is given by:

$$E_{s}=\frac{A^{2}}{2D}\cdot \frac{Q_{0}}{2\pi f_{0}}\cdot \frac{1}{Q_{0}^{2}\left(1-\phi^{2}\right)+\phi^{2}}.$$

The application of the Fourier transform can be considered to be tantamount to referring the behavior of the system to one or the other of two mutually perpendicular planes: The steady-state condition is described by the representation of the state of the system in the energy versus frequency plane and it of necessity deals with the steady state because the variable, time, is not involved. The transient behavior of the system is represented in the energy versus time plane. It is easier to understand that this is orthogonal to the frequency representation if we omit the normalization often used, of choosing the unit of time in terms of the natural period of the system. Nevertheless, it is more convenient to express the behavior of a system in terms relative to its natural parameters, and for the sake of simplicity of equations, much of the discussion will be related to the natural properties of the system.

Three properties serve to specify the behavior of an analyzer described by a second-order differential equation: its undamped natural frequency, *f*_0_, its figure of merit, *Q*_0_, and its “least count,” Δ*E*, the least energy change that can be resolved by the system. The changes in energy content of the system may be thought of as taking place within the signal space bounded by the energy-time and energy-frequency planes, and the representation of the behavior of the system as a function of time or of frequency may be considered as projections upon the principal planes. Since the actual use of the system as an analyzer is never wholly steady-state or completely broadband, the actual process of analysis may be considered as taking place in some plane within the signal space bounded by the principal planes. Depending upon the information sought, the plane of the analyzer will be close to one or the other of the principal planes.

Ordinarily, an analyzer indicates a running time average over the energy, *E_s_*, stored in it. For steady-state signals the indication becomes proportional to the input power; for signals of very brief duration the analyzer responds ballistically and thus gives an indirect indication of energy. The limit of resolution is fixed by the least change in energy storage, Δ*E*, that can be resolved under the circumstances of analysis. In this discussion we shall be dealing with incremental ratios. As the differential will always be considered jointly with the total energy stored over the same time interval, it will be possible in general to discuss ratios of incremental power, Δ*W*, to input power, *W*_0_, or incremental energy, Δ*E*, to energy stored, *E_s_*, interchangeably.

As a function of time, the building up and decay of the energy stored within the system are exponential processes. Therefore it proves convenient to describe the behavior of the system in terms of an exponential variable. Thus, to express the changes in energy storage, we choose an exponential coefficient, *α*. The energy resolution limit, Δ*E*/*E*_0_, may be expressed in terms of *α* through the following definition: If the initial amount of energy stored in the analyzer is *E*_0_, and the minimum change of energy that can be discerned is Δ*E*, then in terms of the exponential variable *α* the equivalent statement is that the energy content must decrease to an amount (*e*^−^*^α^*) times its original value for the change to be at least equal to the minimum change discernible. Expressed as an equation, this limit is given by Δ*E*= (1 — *e*^−^*^α^)E*_0_ for energy, and in the many cases in which we are dealing with energy flow through the system, alternatively as Δ*W *= (1 — *e*^−^*^α^) W*_0_ for power. From the definition of *α* in terms of Δ*E* and *E*_0_, it is evident that:

$$\alpha =-ln\left(1-\frac{\Delta E}{E_{0}}\right).$$

However, it is not along the time axis alone that *α* proves such a convenient function. Because of the close relation between exponential and angle functions, it also yields simple equations for the behavior of the system as a function of frequency. For the foregoing reasons, we shall describe the manner in which the independent variable, the energy increment Δ*E*, influences the resolution limits in frequency and time in terms of the variable *α*, and we will then return to consideration of what the equations describing these resolution limits mean in terms of Δ*E*. We will also discuss special types of noise conditions that may give rise to the irreducible increment that Δ*E* represents.

The conditions under which *α* sets the resolution limit along the time axis are derived from considering the system to contain an amount of energy *E*_0_ at time *t *= 0, and at that instant and for some time subsequent to that, to be free of any driving force. The resolution limit along the time axis is fixed by the least time interval, Δ*t*, during which the system is capable of changing its energy storage by the factor, *e*^−^*^α^*. This corresponds to dissipating at least the discernible energy increment, Δ*E*, during the time Δ*t*. The resolution limit stated in terms of the natural period of the system, *T*_0_, is thus given by Δ*t*/*T*_0_.

When a system of this sort is used as an analyzer, the time interval over which the observation takes place must be of sufficient length for some change to be indicated. Thus the observation interval, Δ*τ*, must equal or exceed the least time interval Δ*t*. (In the previous papers cited in reference 2, the observation interval Δ*τ* was used in place of the least time increment Δ*t*. Use of the least time increment converts several previously found inequalities to equations.)

It is a very close approximation (see appendix 1) to consider only the exponential factor in the decay of energy in the system. The oscillatory terms arising from the sinusoidal nature of the dissipation process are always relatively minor. Thus, regardless of the precise initial conditions,

$${\frac{E}{E_{0}}|}_{\left(t=\Delta t\right)}=e^{-\alpha }\approx \exp\left(-\frac{2\pi }{Q_{0}}\cdot \frac{\Delta t}{T_{0}}\right)$$

and, therefore, the resolution limit along the time axis, expressed in terms of the natural undamped period of the system is

$$\frac{\Delta t}{T_{0}}\approx \frac{\alpha Q_{0}}{2\pi }.$$

From this equation one can see that the ratio of *α* to Δ*t* is twice the real coordinate of the poles of the system on a Nyquist diagram.

The system is selective with respect to the sinusoidal frequency of the driving force which acts as a source for the energy it stores. This makes it suitable for the detection of sinusoidal frequencies falling within its range of response, or a group of similar systems with different *f*_0_’s may be used in the analysis of the components of complex signals. For this purpose, the observation takes place in a plane approximating the energy-frequency plane. Along the frequency axis, one may speak of a frequency resolution limit in this sense: If one knows the natural frequency *f*_0_ to which an analyzer is tuned, then a maximum indication of energy storage for a steady-state sinusoidal signal corresponds to a signal in the vicinity of *f*_0_. Until the energy storage has changed by an amount in excess of Δ*E* or, when expressed as a relative proportion, a factor in excess of Δ*E*/*E*_0_, the departure from maximum indication is not observable. The change in frequency required to produce this effect, Δ*f*, corresponds to the frequency resolution limit Δ*f*/*f*_0_.

As one can see from the equation for the energy stored in the steady-state condition, the response of the system to a sinusoidal frequency other than its natural undamped frequency is diminished to a fraction, *F*, of the maximum energy that would be stored at the natural frequency. Expressed in terms of the ratio of the frequency of the driving force to the natural undamped frequency of the system (*f*/*f*_0_*=ϕ*), the fraction is

$$F=\frac{1}{\phi^{2}+Q_{0}^{2}{\left(1-\phi^{2}\right)}^{2}}.$$

The frequency limits for the region Δ*f* surrounding *f*_0_ are found by solving for the condition *e*^−^*^α^=F*. Solving for the upper and lower frequency limits, *f_b_* and *f_a_*, for which the energy stored in the resonating system is a fraction *e*^−^*^α^* of the peak response yields the expression:

$$\frac{f_{b}^{2}-f_{a}^{2}}{f_{0}^{2}}=\frac{2}{Q_{0}}\sqrt{\left(e^{\alpha }-1\right)+\frac{1}{4Q_{0}^{2}}}.$$

And to a very close approximation, the frequency resolution limit comes out as:

$$\frac{\Delta f}{f_{0}}=\frac{\sqrt{\left(e^{\alpha }-1\right)}}{Q_{0}}$$

when Δ*f* is defined as (*f*_b_—*f_a_*).

The foregoing discussion may now be summarized in geometrical form by reference to a three-dimensional figure in signal space. The limits of resolution of an analyzer may be represented by an irreducible region in a three-dimensional space that must be exceeded before any information about a signal can be found. This space is shown in figure 1. As one can see from the equations, the figure of merit, *Q*_0_, enters into the resolution limits for time and frequency in a complementary way. Thus, it determines the relative proportions between the resolution limits Δ*f*/*f*_0_ and Δ*t*/*T*_0_. One may therefore apportion the relative uncertainties in frequency and time to suit the requirements of an experiment whenever one can control the *Q* of a system. This process is discussed more fully in “Uniform Transient Error” (see footnote 2). But the product of the resolution limits, the “uncertainty equation” depends in irreducible fashion upon *α*.

Thus the signal uncertainty equation, expressed in terms of *α*, turns out to be

$$\Delta f\cdot \Delta t=\frac{1}{2\pi }\sqrt{\left(e^{\alpha }-1\right)}$$

where *α* is related to Δ*E* through the definition of the least change Δ*E* that may be observed in the total stored energy *E*_0_.

The basic form of the resolution equations results from substitution for the exponential coefficient, *α*, in the equations already derived:

$$\frac{\Delta t}{T_{0}}=-\frac{Q_{0}}{2\pi }ln\left(1-\frac{\Delta E}{E_{0}}\right)$$

$$\frac{\Delta f}{F_{0}}=\frac{1}{Q_{0}}\sqrt{\frac{\frac{\Delta E}{E_{0}}}{\left(1-\frac{\Delta E}{E_{0}}\right)}}$$

$$\Delta f\cdot \Delta t=\frac{1}{\pi }\sqrt{\frac{\Delta E}{E_{0}}}\cdot \frac{1}{\sqrt{1-\frac{\Delta E}{E_{0}}}}ln\frac{1}{\sqrt{1-\frac{\Delta E}{E_{0}}}}$$

The volume corresponding to the irreducible limits in signal space, a quantity here defined as the “indication limit,” can be computed from the signal uncertainty equation. It is defined by the triple product of the resolution limits along the three axes,

$$U=\frac{\Delta E}{E_{0}}\cdot \frac{\Delta t}{T_{0}}\cdot \frac{\Delta f}{f_{0}},$$

and it is just the volume of the elementary figure shown in figure 1. Its computation might at first glance appear to be somewhat redundant to the signal uncertainty equation. In fact, its functional form permits factoring the expression for *U* into components that have an interesting connotation in physical measurements.

Thus, since:

$$U=\frac{\Delta E}{E_{0}}\cdot \frac{\Delta f}{f_{0}}\cdot \frac{\Delta t}{T_{0}}\approx \frac{\Delta W}{W_{0}}\cdot \frac{\Delta f}{f_{0}}\cdot \frac{\Delta t}{T_{0}}$$

the magnitude of *U* expressed in terms of the variable, *α*, is

$$U=\frac{\alpha e^{-\alpha }}{2\pi }{\left(e^{\alpha }-1\right)}^{3/2}$$

and *U* is given in terms of the energy resolution limit, Δ*E*/*E*_0_, by:

$$U=\frac{1}{2\pi }\cdot {\left(\frac{\frac{\Delta E}{E_{0}}}{1-\frac{\Delta E}{E_{0}}}\right)}^{3/2}\cdot \left[-\left(1-\frac{\Delta E}{E_{0}}\right)ln\left(1-\frac{\Delta E}{E_{0}}\right)\right].$$

It should be noted that the indication limit *U* and the signal uncertainty relation Δ*f*·Δ*t* depend only upon the energy resolution limit. The energy resolution limit is an independent variable and cannot be reduced by operations altering the function of the system along the *f* and *t* axes: for instance, changing the *Q*_0_ of the system.

For a system operating at its optimum, the irreducible energy or power increment for the system would be set by the noise energy stored in the system. This noise energy may be due to Brownian motion in the system, for example. Noise of external origin may be present with the driving signal. In the general case the intrinsic and extrinsic noise powers will not be coherent, and the total noise energy stored in the system may usually be considered as the sum of contributions.

The prior papers relating the resolution limits to the relative amounts of signal and noise present (see footnote 2) were based upon a derivation subject to the restriction that the signal-to-noise ratio be high. In those papers, the relationship found was

$$\Delta f\cdot \Delta \tau \geq \frac{{\left(S/N\right)}^{-3/2}}{2\pi }$$

where *S* and *N* were signal and noise powers, respectively, and where the use of the observation interval Δ*τ* rather than the limiting time increment Δ*t* made the relation an inequality.

Such a restriction was, in fact, not required. A tractable and useful expression for the product Δ*f*·Δ*t* can be derived, valid for all values of *S*/*N*.

In the case where both signal and noise energy are stored, the total energy present in the system is *E*_0_*=S*+*N*. An increment in the signal energy stored (or in the input signal power) can be detected only if it equals or exceeds the minimum energy increment the system is capable of indicating. From this definition and the definition of the exponential factor, *α*:

$$\frac{\Delta S}{S+N}\geq \frac{\Delta E}{E_{0}}=\frac{\Delta W}{W_{0}}=\left(1-e^{-\alpha }\right).$$

And, where the least energy increment is controlled by the noise energy stored; Δ*E→N:* or, very nearly

$$\frac{\Delta E}{E_{0}}=\frac{N}{S+N}.$$

Thus, where the limit of detection is set by the noise energy:

$$\alpha =ln\frac{N+S}{S}$$

from which the signal uncertainty becomes:

$$\Delta f\cdot \Delta t=\frac{1}{2\pi }\sqrt{\frac{N}{S}}\cdot ln\left(1+\frac{N}{S}\right).$$

For high values of *S*/*N*, this expression approaches the limit (1/2π) (*S*/*N*) ^−3/2^, a result found previously (see footnote 2).

An especially interesting interpretation can be made from the form of the expression for the indication limit, *U*, when it is written in terms of the signal-to-noise ratio.

$$U=\frac{{\left(S/N\right)}^{-3/2}}{2\pi }\cdot \left(\frac{-S/N}{1+S/N}ln\frac{S/N}{1+S/N}\right).$$

The first factor can be recognized to be the expression for the signal uncertainty, Δ*f*·Δ*t*, when the signal-to-noise ratio is high. The term in parentheses also has a recognizable functional form, and in fact it is possible to relate it to the limiting probability of information transfer.

To facilitate discussion, the factor *N* may be cleared from the fractions in the term, giving it the form: *—S*/*(S+N) l*n *S*/*(S+N)*. As one can see from the series expansion for the natural logarithm, this product approaches the value *N*/*S* for large values of *S* relative to *N*, and the indication limit then is merely the trivial product of the reciprocal of the signal-to-noise ratio multiplied by the signal uncertainty function. It is quite another matter as *S*/*N→*0. Then the indication limit may be considered to consist of two meaningful terms: one is the same signal uncertainty function that has already been derived for high signal-to-noise ratios (see footnote 2) (i.e., for Δ*E* small *re E*_0_); the other is a modulating function that we will now proceed to relate to statistical matters.

Where one is dealing with the statistical presence of noise and signal, the long-time average signal-tonoise ratio may be described in terms of expected values. The following definition of expected value is taken from a textbook on statistics.^5^ “The expected value of a random variable or any function of a random variable is obtained by finding the average value of the function over all possible values of the variable…. This is the *expected value*, or *mean value* of *x*. It is clear that the same result would have been obtained had we merely multiplied all possible values of *x* by their probabilities and added the results … we might reasonably expect the average value of *x* in a great number of trials to be somewhere near the expected value of *x.”*

A special case that is quite common experimentally is one in which the level distributions of signal and noise are precisely the same. If one has either a signal or noise, the probability of the signal being *P*, then it follows that the probability of the noise is (1–*P*). If the signal and noise have the same level distribution, *G*, the modulating term

$$\frac{S}{N+S}ln\frac{S}{N+S}\to \frac{P\cdot G}{P\cdot G+\left(1-P\right)\cdot G}ln\frac{P\cdot G}{P\cdot G+\left(1-P\right)\cdot G}$$

and thus the term that modulates the signal uncertainty function can be seen to reduce to the form —*PlnP*, where *P* is the probability of signal occurrence. From very simple considerations, therefore, the limit of detection is shown to be related directly here to a limiting probability of information transfer—a quantity usually derived in information theory by considering signals and noise to be made up of equal-sized unit impulses.

It is interesting that this point was arrived at in the reverse direction by Woodward and Davies.^6^ They started with the *PlnP* term from Shannon’s information function and demonstrated from considering the signals and noise in radar detection that the quantity *P* was related to the signal-to-noise ratio for radar signals.

However, the modulating term in its original form, in terms of *S* and *N*, may be seen to represent a generalization of the function defined in information theory as the channel capacity, *H*. This form is more closely related to the ordinary formulation describing the entropy of a system in terms of the probability distribution of energy states within it.^7^

The question of whether a signal is detected in the presence of noise depends upon what the investigator chooses to consider a reasonable limiting probability in deciding whether a signal has been detected. A preponderance of only 1 percent above random distribution would correspond to a much smaller signal-to-noise ratio than would 90 percent. In fact, the common definition that gives the limit of detection as a signal-to-noise ratio of unity corresponds directly to setting the criterion for the limiting probability of detection at 50 percent.

For the case *N=S*, the *“—PlnP”* term becomes $-\frac{1}{2}$ *ln* $\frac{1}{2}$ and the indication limit becomes:

$$U_{1}=\frac{1}{2\pi }\left(-\frac{1}{2}ln\frac{1}{2}\right)=\frac{ln2}{4\pi }$$

for which the signal uncertainty function is:

$$\Delta f\cdot \Delta t=\frac{ln2}{4\pi }.$$

These are the limiting forms also where signals and noise are transmitted as “bits.”

Thus far we have discussed noise from the standpoint of the noise energy stored in the system. This energy is, so far as the actual storage in the system is concerned, not distinguishable from energy stored that might be derived from purely sinusoidal excitation. If all excitation were withdrawn, and the system left in the force-free state, the energy in it would be dissipated in the usual exponential decay, and the oscillations during the decay would be essentially of frequency *f*_0_, providing the system had moderate energy storage capacity (*Q*_0_>1).

Therefore, unless we have some other means for distinguishing among the sources of the energy stored in the system—such as, for example, knowledge of the spectral character of pulsed signals applied to the system—or knowledge of the amount of energy found present in the system when it is considered to be free of any known source, we are left to regard as signal that part of the energy stored in the system that was produced by a sinusoidal signal of power *S_i_*. The remaining driving sources, of more or less broad spectral distribution, would in general be classified as noise.

If the system is being driven at its natural frequency, its output signal will be *Q*_0_/2π times its input, for *Q*_0_ is 2π times the ratio of the energy stored to the energy dissipated during the cycle. At any other driving frequency, the energy stored will be weighted by the response, *ρ*, which relates the energy stored to the power supplied to the system.

$$\rho =\frac{Q_{0}/2\pi f_{0}}{f^{2}/f_{0}^{2}+Q_{0}^{2}{\left(1-f^{2}/f_{0}^{2}\right)}^{2}}.$$

Suppose the noise within the system arises from a source whose spectral distribution is given by the power density function, *N_f_*. The system will store energy with a weighting factor of *ρ*.

The total energy stored in the system due to excitation by noise will then be found from the integral:

$$N=\int_{0}^{\infty }N_{f}\rho d\omega .$$

Although we have discussed the behavior of the system in terms of cycles per second, the quantity we have designated as *Q*_0_ is defined directly in terms of the ratio of the energy stored to the energy dissipated *per radian*. As the parameter of the function we are integrating is *Q*_0_, we must choose the dimensionally similar variable in order to carry out the integration correctly. Thus we must integrate with respect to *ω* rather than *f*. This point is given in detail because it is an instance of the dimensionality of angles recently pointed out by C. H. Page.^8^

This integral may be evaluated easily for noise of constant energy per unit bandwidth in cycles per second; the result is then^9^

$$N=\frac{\pi N_{f}}{2}$$

and it is independent of *Q*_0_ because the energy storage capacity of the system and its bandpass for noise are affected in a complementary fashion by changes in the figure of merit.

For several other types of noise, an approximate equivalent white noise coefficient, *n_f_*, can be defined, for which the foregoing equations remain applicable. Given a noise whose spectral distribution is *v(f)*, a mean value “equivalent” white noise per unit bandwidth may be computed from the equation:

$$n_{f}=\frac{\int_{f_{a}}^{f_{b}}v\left(f\right)df}{\int_{f_{a}}^{f_{b}}df}.$$

Obviously if *v(f*) equals a constant, then *n_f_* is the familiar “White Noise” coefficient. However, for several other types of noise the mean-value integration yields an equivalent *n_f_*, which may be treated as a constant, with rather low residual error resulting from this approach. This can be seen from the fact that the system is selective with respect to the frequency components of the power sources from which it stores energy. Thus the restriction is only that the spectral distribution be changing *slowly* in the frequency region immediately surrounding *f*_0_. For the following noise distributions, the residual terms discarded amount, in the worst case, to no more than 12½ percent of the approximate value:

For the noise distributions shown in the left-hand column, the respective mean values are shown in the right-hand column:

$$v\left(f\right)=k\cdot f\;n_{f}=\frac{k}{2}\left(f_{b}+f_{a}\right)$$

$$\underset{\left(\text{constant}\;\text{energy}/\text{octave}\right)}{v\left(f\right)=k/f}\;n_{f}=\frac{k\ln\;f_{b}/f_{a}}{\left(f_{b}-f_{a}\right)}$$

where, for this purpose, *f_b_* and *f_a_* are taken as the upper and lower half-energy limits of the response weighting function, *ρ*.

One can use the foregoing to predict the ratio of energy stored from the sinusoidal excitation to the energy stored from the source of noise. Thus the effective signal-to-noise ratio in terms of energy stored in the system is given by:

$$S/N=\frac{2S_{i\rho }}{\pi n_{f}f_{0}}.$$

The signal-to-noise ratio will be a maximum if the frequency of the sinusoidal excitation is just equal to the natural undamped frequency of the system. (For this condition, *ρ = Q*_0_/2*π*.) The maximum ratio is:

$$S_{0}/N=\frac{S_{i}Q_{0}}{\pi^{2}n_{f}f_{0}}.$$

The foregoing derivation has meaning also with respect to any system described by a second-order differential equation in which some event of brief duration occurs. A quantity directly analogous to the indication limit may be computed. In this instance, the expressions relate the resolution limits that bound the occurrence of the event, specifying the least increments of energy, frequency and time within which the event can take place.

Further, the limiting energy increment, Δ*E*, need not be formed by noise. For example, in a pulse- height system it would correspond to the smallest step in pulse height that might be indicated. As another example, an atomic system described by a second-order differential equation might have Δ*E* subject to quantum limitations.

## Summary

The incremental limits, resolution limits, and indication limit for the performance of any system that is described by a second-order differential equation may be computed from considering the manner in which the system stores and dissipates energy, subject only to the restriction that the system be capable of storing oscillatory energy reversibly. As these limits are inherent in the mathematical properties of second-order differential equations, they are applicable to other types of systems by analogy.

The results of the discussion presented here are summarized in table 1. The relations tabulated apply to conditions that may be observed so long as the observation interval exceeds ¼π times the natural period of the system. The first line of table 1 summarizes the equations in terms of the basic energy resolution limit, Δ*E*/*E*_0_. The second line gives the same results expressed as functions of the exponential quantity *α*, and its inherent advantage as a means of expressing Δ*E*/*E*_0_ can be seen from the greater simplicity of the equations in *α*. The third line summarizes the equations for the condition that Δ*E*/*E*_0_ is limited by the noise energy stored with the signal. The last line is the formulation of the noise- limited case in terms of the input powers of sinusoidal signal and noise, where the noise power can be expressed as noise energy per cycle.

The derivations sketched in the text are presented in detail in a series of appendices for those cases where it is felt this presentation will prove informative.

It is not feasible to relate each idea in this paper to a specific item in the literature. However, there are numerous papers that bear some relation to the material in this paper. A classified bibliography of some of these papers is therefore included at the end of this paper for the convenience of persons working on related problems. (The reader may also wish to consult the bibliography in Pimonow’s book, see footnote 1.)

In this work I have benefited greatly by numerous discussions with Chester H. Page and Richard K. Cook. For several very enlightening remarks I am grateful to William H. Huggins and C. G. M. Fant. For a very helpful discussion on the dimensional properties of *Q*, and for painstaking checking of derivations, I should like to acknowledge the help of my colleague, Joseph Tant Priestley. To a number of my colleagues, friends, and visitors who have sat patiently discussing this problem with me while I was working it out, I should like to take this opportunity of extending my thanks.

This work was sponsored by the Office of Naval Research and also supported in part by the National Bureau of Standards.

## Figures and Tables

**Figure 1:**
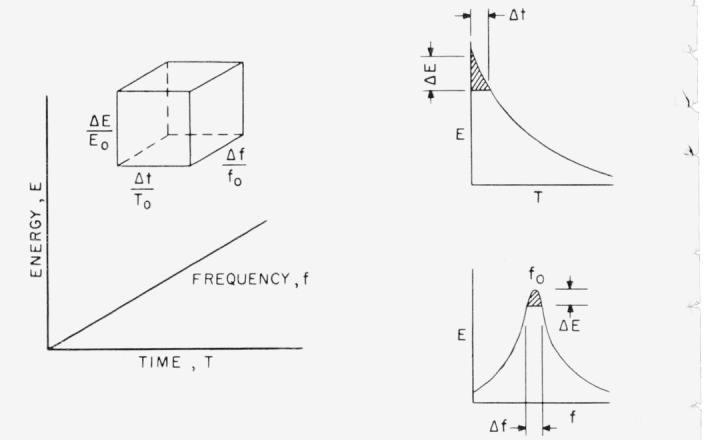
Resolution limits making up the indication limit, *U*. *T_0_* is the period corresponding to *f*_0_, the natural undamped frequency of the system. *E*_0_ is the maximum sinusoidal energy stored.

**Figure 2:**
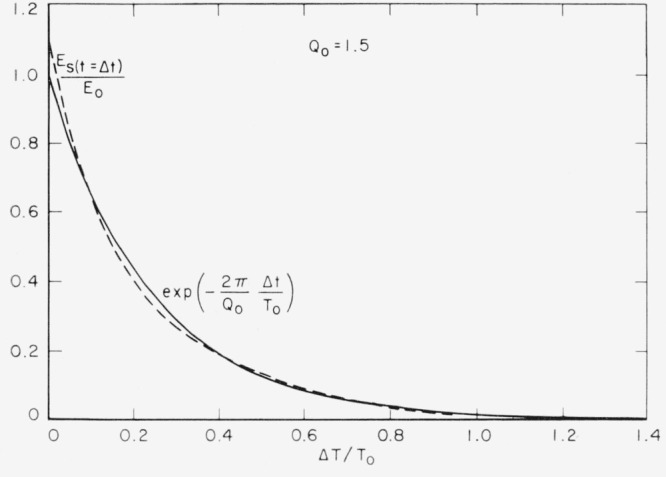
Decay of the energy in an oscillatory system for *Q* of 1½ It is these minor variations that we omit by working only with the exponential decay. They are too minor to represent for values of *Q* in excess of 2.

**Figure 3:**
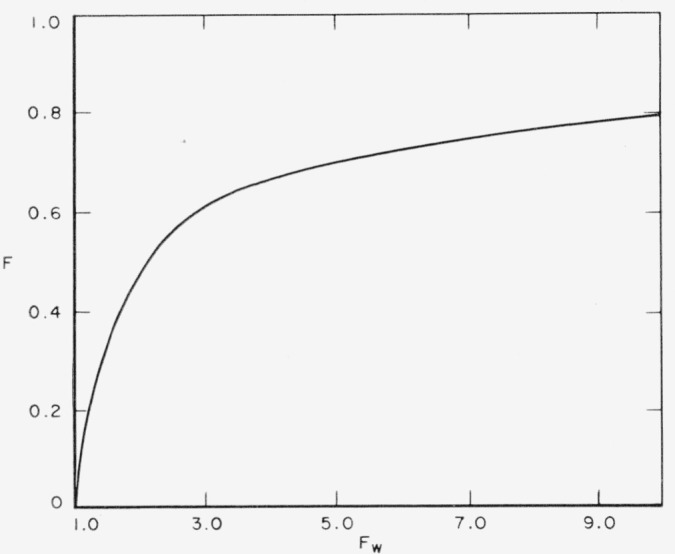
Fraction of total energy stored for various relative response limits.

**Table 1:** Resolution limits of analyzers and oscillatory systems expressed in several alternative variables.

Basic Variables:			Resolution Limits:	

$\frac{\Delta E}{E_{o}}$	α	S/N	Δf/f_0_	Δt/T_0_

$\frac{\Delta E}{E_{o}}$	$-\ell n\left(1-\frac{\Delta E}{E_{o}}\right)$	$\frac{1-\frac{\Delta E}{E_{o}}}{\frac{\Delta E}{E_{o}}}$	$\frac{1}{Q_{o}}\sqrt{\frac{\frac{\Delta E}{E_{o}}}{1-\frac{\Delta E}{E_{o}}}}$	$-\frac{Q_{o}}{2\pi }\ell n\left(1-\frac{\Delta E}{E_{o}}\right)$
$1-e^{-\alpha }$	α	$\frac{1}{e^{\alpha }-1}$	$\frac{\sqrt{e^{\alpha }-1}}{Q_{o}}$	$\frac{\alpha \;Q_{o}}{2\pi }$
$\frac{1}{S/N+1}$	$\ell n\frac{S/N+1}{S/N}$	S/N	$\frac{1}{Q_{o}\sqrt{S/N}}$	$\frac{Q_{o}}{2\pi }\ell n\frac{S/N+1}{S/N}$
$\frac{\pi^{2}n_{f}f_{o}}{S_{i}Q_{o}+\pi^{2}n_{f}f_{o}}$	$\ell n\left(1+\frac{\pi^{2}n_{f}f_{o}}{S_{i}Q_{o}}\right)$	$\frac{S_{i}Q_{o}}{\pi^{2}n_{f}f_{o}}$	$\frac{1}{Q_{o}}\sqrt{\frac{\pi^{2}n_{f}f_{o}}{S_{i}Q_{o}}}$	$\frac{Q_{o}}{2\pi }\ell n\left(1+\frac{\pi^{2}n_{f}f_{o}}{S_{i}Q_{o}}\right)$

Uncertainty Relation:	Indication Limit:

Δf Δt	U

$\frac{1}{\pi }\sqrt{\frac{\Delta E}{E_{o}}}\cdot \frac{1}{\sqrt{1-\frac{\Delta E}{E_{o}}}}\ell n\frac{1}{\sqrt{1-\frac{\Delta E}{E_{o}}}}$	$\frac{1}{2\pi }{\left(\frac{\frac{\Delta E}{E_{o}}}{1-\frac{\Delta E}{E_{o}}}\right)}^{3/2}\left(-\left(1-\frac{\Delta E}{E_{o}}\right)\ell n\left(1-\frac{\Delta E}{E_{o}}\right)\right)$
$\frac{\alpha }{2\pi }\sqrt{e^{\alpha }-1}$	$\frac{\alpha \;e^{\alpha }}{2\pi }{\left(e^{\alpha }-1\right)}^{3/2}$
$\frac{1}{2\pi \sqrt{S/N}}\ell n\frac{S/N+1}{S/N}$	$\frac{{\left(S/N\right)}^{-3/2}}{2\pi }\cdot \left(-\frac{S/N}{S/N+1}\ell n\frac{S/N}{S/N+1}\right)$
$\frac{1}{2\pi }\sqrt{\frac{\pi^{2}n_{f}f_{o}}{S_{i}Q_{o}}}\ell n\left(1+\frac{\pi^{2}n_{f}f_{o}}{S_{i}Q_{o}}\right)$	$\frac{1}{2\pi }{\left(\frac{\pi^{2}n_{f}f_{o}}{S_{i}Q_{o}}\right)}^{3/2}\cdot \left(-\frac{S_{i}Q_{o}}{\pi^{2}n_{f}f_{o}+S_{i}Q_{o}}\ell n\frac{S_{i}Q_{o}}{\pi^{2}n_{f}f_{o}+S_{i}Q_{o}}\right)$

## Footnotes

1 D. Gabor, Theory of communication, J. Inst., Elec. Eng. 93, Part III (1946), 429–457; A. A. Kharkevich, Spectra and Analysis, Translated and published by Consultants Bureau, New York (1969); L. Brillouin, Science and Information Theory, 2d edition, published by the Academic Press, New York (1962); L. Pimonow, Vibrations en Régime Transitoire, Dunod, Paris (1962). (This book has a very extensive bibliography.)

2 Limitations on rapid signal analysis, J. Wash. Acad. Sci. 45, 359–360 (1955); Transients in signal analysis, J. Wash. Acad. Sci. 46, 305–307 (1956); Uniform transient error, J. Research NBS 61, 25–30 (1958) RP2879.

3 The term “least count” is chosen here by analogy to a term describing the limitation in a metering system; the least increment that can be read on the meter.

4 M. Greenspan, Simple derivation of the Boltzmann-Ehrenfest adiabatic principle, J. Acoust. Soc. Am. 27, 34–35 (1955). The adiabatic principle states that for a system excited by a change in parameters, the ratio of energy content to resonant frequency is a constant.

5 A. M. Mood, Introduction to the Theory of Statistics, p. 91. (McGrawHill Book Co., New York, N.Y., 1950).

6 P. M. Woodward and I. L. Davies, A theory of radar information, Phil. Mag. Series 7, 41, 1001–1017 (1950).

7 Max Born, Natural Philosophy of Cause and Chance, The Clarendon Press, Oxford (1949), cf, especially the discussions of probability and entropy; Claude Shannon and Warren Weaver, The Mathematical Theory of Communication, University of Illinois Press. Urbana, Ill. (1949).

8 C. H. Page, Physical entities and mathematical representation, J. Research NBS 65B (Math, and Math. Phys.) No. 4, 227–235 (1961).

9 D. Bierens de Haan, Nouvelles Tables D’Intégrales Définies, eq (6) p. 47 (Edition 1867, reprinted 1939).

## Appendix 1. Energy Storage and Dissipation

For a system whose properties are described by a second-order differential equation, subjected to a driving force of amplitude *A*, the differential equation becomes:

$$M\;\ddot{x}+D\;\dot{x}+kx=A\;e^{j\omega t}.$$

The equation may be rewritten in terms of parameters related to the undamped natural frequency and figure of merit of the system. The relative frequency, *ϕ*, replaces the quantities *f* and *ω* by *ϕf*_0_ and *ϕω*_0_ respectively. The equation resulting from this substitution is

$$\frac{Q_{0}}{\omega_{0}}\ddot{x}+\dot{x}+\omega_{0}Q_{0}x=\frac{A}{D}e^{j\phi \;\omega_{0}t},$$

where, of course, the ratio *k*/*M* is *ω*_0_^2^.

The system responds to the force by motion of amplitude *B*, whose phase relative to *A* is given by the angle θ.

$$\theta =\tan^{-1}\frac{\phi }{Q_{0}{\left(1-\phi \right)}^{2}}$$

and the absolute value of the square of the amplitude is

$$\left|B^{2}\right|=\frac{A^{2}}{{\omega_{0}}^{2}D^{2}}\cdot \frac{1}{{Q_{0}}^{2}{\left(1-\phi^{2}\right)}^{2}+\phi^{2}}.$$

The conventional definition for the quantity we represent here by *Q*_0_ is given in terms of the natural undamped period of the system. For a sinusoidal driving force, one may have more use for the energy dissipated per cycle of the driving force, and in general the exciting force will not necessarily be alternating at the natural frequency of the system.

We wish to extend the definition of *Q* for a system when it is being excited at frequencies other than its natural frequency. There are several alternative definitions ordinarily given for the quantity we designate here as *Q*_0_.

$$\begin{array}{l} Q_{0}=\frac{\text{Peak}\;\text{energy}\;\text{stored}}{\text{Mean}\;\text{energy}\;\text{dissipated}\;\text{per}\;\text{radian}\;\text{fraction}\;\text{of}\;\text{the}\;\text{natural}\;\text{period}}\\ \;=2\pi \frac{\text{Peak}\;\text{energy}\;\text{stored}}{\text{Energy}\;\text{dissipated}\;\text{per}\;\text{cycle}\;\text{of}\;\text{the}\;\text{natural}\;\text{period}}\\ \;=\omega_{0}\frac{\text{Peak}\;\text{energy}\;\text{stored}}{\text{Mean}\;\text{rate}\;\text{of}\;\text{power}\;\text{dissipation}} \end{array}$$

The second definition is commonly given because the amount of energy dissipated in a single cycle can be computed without finding a mean value. For our purposes, the third alternative gives the most direct approach to a generalized definition of *Q*.

The peak energy stored in the system under steady-state excitation is given by:

$$E_{s}=\frac{A^{2}}{2D}\cdot \frac{M}{D}\cdot \frac{1}{{Q_{0}}^{2}{\left(1-\phi^{2}\right)}^{2}+\phi^{2}}$$

and the mean rate of power dissipation is

$${\bar{W}}_{D}=\frac{A^{2}}{2D}\cdot \frac{\phi^{2}}{{Q_{0}}^{2}{\left(1-\phi^{2}\right)}^{2}+\phi^{2}}.$$

The term ω_0_ in the third alternative of the definition for *Q*_0_ may be rewritten as 2π/*T*_0_, where *T*_0_ is the natural period of the system.

Let us denote by *Q_f_* the ratio of peak energy stored to the energy dissipated during one cycle of the driving frequency. The period of the driving frequency is *T=T*_0_/*ϕ* and the resultant equation for *Q_f_* becomes

$$Q_{f}=Q_{0}/\phi .$$

However, when a system is subjected to excitation by a complex group of driving forces, the only consistent period over which to compute the energy dissipated is the natural period of the system. For one frequency component of the complex signal, whose frequency is *ϕ* times the natural frequency of the system, the system stores and dissipates energy during excitation as though it had a *Q* given by:

$$Q_{\phi }=\frac{Q_{0}}{\phi^{2}}.$$

Note that either of these more general definitions of *Q* reverts to *Q*_0_ for excitation at the natural frequency of the system. These expressions show that the ratio of energy stored by the system to the energy dissipated is a rather smooth function of frequency. The familiar frequency-selective action of the system is in fact shown by the amount of energy stored in the system under excitation by a sinusoidal force of a given *amplitude*, and is a consequence of the material lowering of impedance in the vicinity of the resonant frequency of the system.

The exponential decay of the energy stored in the system after cessation of the driving force takes place at a frequency very near to the natural undamped frequency of the system. Owing to the dissipation, the frequency is lowered by the factor

$$\delta =\sqrt{1-\frac{1}{4{Q_{0}}^{2}}}.$$

Thus the time scale of the decay is most closely expressed by the figure of merit, *Q*_0_.

The exponential factor *α* has been defined from the exponential envelope of the decay of the energy stored in the system after the driving force was withdrawn. This is an approximation in that no account is taken of the instantaneous phase of the energy stored in the system at the instant when the driving force ceased. In fact, this is no gross approximation, as one can see by inspection of the equation that describes the process. Letting *x_a_* and *x_b_* represent the amplitudes of the sine and cosine terms representing the coordinates of the system at *t*= 0:

$$\begin{array}{r} E_{s}=\frac{\pi Q_{0}D}{T_{0}}\cdot e^{-\frac{2\pi }{Q_{0}}\cdot \frac{t}{T_{0}}}\{\left(x_{a}^{2}+x_{b}^{2}\right)+\frac{1}{4Q_{0}}\left[\frac{x_{a}^{2}-x_{b}^{2}}{Q_{0}}-4\delta x_{a}x_{b}\right]\\ \cos\frac{4\pi \delta t}{T_{0}}+\frac{1}{4Q_{0}}\left[\frac{x_{a}x_{b}}{Q_{0}}+4\delta \left(x_{a}^{2}-x_{b}^{2}\right)\right]\sin\frac{4\pi \delta t}{T_{0}}\}. \end{array}$$

As the first term is the square of the modulus of the system coordinate at *t*=0, it is independent of time. The remaining terms, which form the modulus of a double-frequency component, represent minor variations from exponential decay of the energy stored in the system. The main term is a sum of squares, whereas the modulus of the double-frequency component contains the difference of $x_{a}^{2}$ and $x_{b}^{2}$ and the product *x_a_x_b_*. Neither the difference nor the product can exceed the sum of the squares, and they are further diminished by a factor of 1/*Q*_0_ or smaller, depending upon the initial conditions. The magnitudes of the double-frequency terms depend upon the conditions at *t*=0, but they are always fairly unimportant. The situation is shown graphically in figure 2.

The discussion can be carried one step further: As the energy in a force-free system can only be dissipated, *dE_s_*/*dt* is not positive during any part of the decay. Therefore, the slope of the modulated exponential curve is never greater than zero.

## Appendix 2. Noise

Noise can be considered as the result of a number of sinusoidal components acting simultaneously. The steady-state storage of noise energy in the system can be computed because the energy dissipated will be equal to the energy supplied.

Usually, when we speak of noise of constant power per unit bandwidth, we imply that the driving force amplitude is described by the equation:

$$\frac{A^{2}}{2D}=n_{f}\cdot f.$$

Since the power dissipation in the system at its resonant frequency is given by $W_{D}=A_{0}^{2}/2D$, the parameter *n_f_* has the dimensions of energy per cycle.

At the frequency of resonance for the system, the energy stored is

$$E_{0}=\frac{Q_{0}}{2\pi f_{0}}\cdot n_{f}f_{0}.$$

For a driving force of a given magnitude, the peak energy stored by the system at any frequency is related to the peak energy stored at the frequency of its natural resonance by the fraction, *F*:

$$F=\frac{1}{{Q_{0}}^{2}{\left(1-\phi^{2}\right)}^{2}+\phi^{2}}.$$

The total noise energy stored is found by integrating over this function. The parameter *Q*_0_ is defined for energy dissipated per radian. Therefore the weighting for the noise energy stored is

$$\int_{0}^{\infty }E\cdot F\;d\omega .$$

The integration may be stated in terms of the variable, *ϕ*, by making the substitution *dω* = ω_0_*dϕ *= 2*πf*_0_*dϕ*. After some rearrangement of terms, the noise energy stored in the system is given by:

$$N=\frac{1}{Q_{0}}\int_{0}^{\infty }\frac{n_{f}d\phi }{1+2\left(\frac{1}{2Q_{0}^{2}}-1\right)\phi^{2}+\phi^{4}}.$$

Under circumstances for which *n_f_* may be removed from the integration, the remainder of the integral becomes merely (*πQ*_0_)/2, so that *N *= (*πn_f_*)/2. For a sinusoidal input signal of average power ${\bar{W}}_{s}$, at the resonance frequency of the system, the maximum ratio of signal energy stored to noise energy stored is given by:

$$S/N=\frac{Q_{0}{\bar{W}}_{s}}{\pi^{2}n_{f}f_{0}}.$$

In general, *n_f_* would be some function of frequency *v(f*), and the evaluation of the noise weighting integral would be more difficult. However, since the systems we are dealing with are somewhat selective, there are some other noise distributions for which a useful “equivalent white noise coefficient” can be approximated. We have defined an equivalent white noise coefficient as the mean value of the noise function over the passband of the system.

For the type of noise that may be described by *v(f)=kf* the mean-value integration gives:

$$n_{f}=\frac{k}{2}\left(f_{b}+f_{a}\right).$$

The range of values of *v(f*) is *k(f_b_—f_a_*) whereas the “equivalent” *n_f_* is *(k*/2) (*f_b_*+*f_a_*). The ratio of the range of values for *v(f*) to the computed value for *n_f_* represents an extreme estimate for the error made in using *n_f_*. It represents the situation for a flat weighting of the noise components, whereas the system is actually selective and gives most weight to components in the vicinity of *f*_0_. This extreme estimate yields the ratio:

$$\frac{v\left(f_{b}\right)-v\left(f_{a}\right)}{n_{f}}=\frac{2\left(f_{b}/f_{a}-1\right)}{\left(f_{b}/f_{a}+1\right)}.$$

For systems with a *Q* of 3 or higher, the response of the system falls away rapidly from the peak response, and the ratio *f_b_/f_a_* is very nearly unity when *f_a_* and *f_b_* are taken as the one-half power points. For the half-power points at *Q *= 3 this error estimate represents the fraction 1/3, but half the total integrated energy stored in the system is stored within the range of frequencies *between f_a_* and *f_b_*; the other half arises from the remainder of the frequency range between zero and infinity.

(See appendix 3 for derivation of the energy stored within restricted ranges of frequency.)

For *v(f)=k*/*f*, the equivalent white noise concentration, *n_f_*, is given by:

$$n_{f}=\frac{k\;\ln\;f_{b}/f_{a}}{f_{b}-f_{a}}.$$

It is necessary here to apply l’Hôpital’s Rule to see that this equation is valid even for high values of *Q* because as *f_b_→f_a_*, it simply approaches the limit *n_f_=k*/*f_a_*. Thus, as it should, the mean value, *n_f_*, approaches the value *v(f*_0_), as the range over which the mean value is computed decreases.

For low values of *Q*, the question of the error introduced by substituting the mean value for the actual function is again limited at worst to the ratio that the range of values of *v(f*) bears to the mean value *n_f_*. Thus, for the function *v(f)=k/f*.

$$\frac{v\left(f_{b}\right)-v\left(f_{a}\right)}{n_{f}}=\frac{{\left(f_{b}/f_{a}-1\right)}^{2}}{\frac{f_{b}}{f_{a}}\ln\frac{f_{b}}{f_{a}}}.$$

Because of the relatively slow change of the logarithm as a function of its argument, this range always represents a fairly small error.

In summary, we have shown that an equivalent white noise figure can be derived from the mean value of several noise functions, and that substitution of an *n_f_* derived from the mean value of *v(f)* in place of the white noise constant does not lead to serious error. Thus the relations derived in this paper for ordinary white noise are applicable to a number of other noise functions as well.

## Appendix 3. Distribution of Energy Versus Frequency, and the Noise Integral

By patterning the integration upon the polynomial used in equations 6 and 7 on page 47 in the tables of Bierens de Haan, we can compute the fraction of the total noise power taken up by the system between any pair of fractional-power points in the frequency range, and the fraction of the total noise energy that is stored within the frequency range bounded by any pair of fractional energy points.

For the system, the mean rate of power dissipation is given by:

$${\bar{W}}_{D}=\frac{\frac{A^{2}}{2D}\phi^{2}}{Q_{0}^{2}{\left(1-\phi^{2}\right)}^{2}+\phi^{2}}$$

The energy stored is given by:

$$E_{s}=\frac{\frac{A^{2}}{2D}\cdot \frac{Q_{0}}{2\pi f_{0}}}{Q_{0}^{2}{\left(1-\phi^{2}\right)}^{2}+\phi^{2}}$$

At the natural resonance frequency of the system, *ϕ*^2^= 1, and thus, when expressed as a fraction of the response at the natural frequency, the relative power and relative energy functions become independent of the driving force. Choosing for convenience to express the fractional response as a reciprocal (e.g., 2 for the one-half power, etc.) for fractional energy,

$$F_{E}=Q_{0}^{2}{\left(1-\phi^{2}\right)}^{2}+\phi^{2}$$

and, for fractional power,

$$F_{W}=\frac{Q_{0}^{2}{\left(1-\phi^{2}\right)}^{2}+\phi^{2}}{\phi^{2}}.$$

One can then solve these equations to find at what relative frequency limits the system has a given fractional response. This procedure yields, for the frequency range between the fractional energy points, the *approximate* result:

$$\frac{\;f_{b}-f_{a}}{f_{0}}=\frac{\sqrt{F_{E}-1+1/4Q_{0}^{2}}}{Q_{0}}$$

and, for the frequency range between the fractional power points, the *exact* solution:

$$\frac{\;f_{2}-f_{1}}{f_{0}}=\frac{\sqrt{F_{W}-1}}{Q_{0}}.$$

The approximation needed in solving for the fractional energy points arises from the fact that the system has finite energy storage capacity at zero frequency, whereas it has zero energy storage capacity at infinite frequency. The power dissipation capacity of the system is, however, zero at both extremes of the frequency range, and the function of power dissipation versus the logarithm of the frequency is symmetrical. Except for systems of low energy-storage capacity, the asymmetry of the energy storage function is negligible. (Its influence on Δ*f*/*f*_0_ is discussed in appendix 4.)

Within the frequency range between any two fractional energy limits, the noise energy stored is given by:

$$N_{E}=\frac{n_{f}}{Q_{0}}\int_{\phi_{a}=f_{a}/f_{0}}^{\phi_{b}=f_{b}/f_{0}}\frac{d\phi }{1+2\left(\frac{1}{2Q_{0}^{2}}-1\right)\phi^{2}+\phi^{4}}.$$

This integral can be divided into partial fractions, and the separated integrals give angle functions, from which the combined result is

$$N_{E}=\frac{n_{f}}{Q_{0}}\cdot \frac{Q_{0}}{2}\tan^{-1}\frac{-2\sqrt{F_{E}-1+1/4Q_{0}^{2}}}{\left(F_{E}-1\right)\sqrt{1-F_{E}/Q_{0}^{2}}}$$

The radical in the denominator becomes imaginary for the condition in which the energy storage at zero frequency exceeds the value of *F_E_* chosen for the frequency limit.

The power taken up by the system between the frequency limits representing various fractional power points is given by the integral:

$$\begin{array}{c} W_{f}=\frac{2\pi n_{f}f_{0}}{Q_{0}^{2}}\int_{\phi_{1}=f_{1}/f_{0}}^{\phi =f_{2}/f_{0}}\frac{\phi^{2}d\phi }{1+2\left(\frac{1}{2Q_{0}^{2}}-1\right)\phi^{2}+\phi^{4}}\\ =\frac{\pi n_{f}f_{0}}{Q_{0}}\cdot \tan^{-1}\frac{2\sqrt{F_{W}-1}}{2-F_{W}} \end{array}$$

where *ϕ*_1_ and *ϕ*_2_ are the limits of relative frequency corresponding to the fractional powers whose reciprocal is *F_W_*. This equation requires no approximation.

The graphical result of this integration is shown in figure 3. The stored energy derived from noise power between the frequency limits representing various fractional power points is presented as a fraction of the total storage for the complete spectrum.

These integrals between finite limits may also be applied to evaluating the storage and dissipation of energy derived from band-limited sources.

## Appendix 4. Frequency Resolution Limit

The approximation that leads to computation of Δ*f*/*f*_0_ has interesting connotations in physical problems. One of the terms discarded represents an asymmetry in the function describing the energy response of the system as a function of frequency. This term becomes appreciable only for systems of low energy-storage capacity $\left(Q\to \frac{1}{2}\right)$, but its computation permits a quantitative estimate to be made as to the limits within which the equations derived in this paper are applicable.

The boundaries of the frequency resolution limit, Δ*f*/*f*_0_, are those for which the energy storage is less than the maximum steady-state storage by the limiting energy increment, Δ*E*. This condition may be found from solutions of the equation:

$$e^{-\alpha }=\frac{1}{Q_{0}^{2}{\left(1-\phi^{2}\right)}^{2}+\phi^{2}}.$$

A convenient method is to rewrite the equation as:

$${\left(\phi^{2}-1\right)}^{2}+\left(\phi^{2}-1\right)/Q_{0}^{2}-\left(e^{\alpha }-1\right)/Q_{0}^{2}=0$$

and to solve for the quantity *(ϕ*^2^*—*1). The two roots can be written as

$$\begin{array}{l} d_{1}=\left(\phi_{1}^{2}-1\right)=\left(f_{1}/f_{0}-1\right)\left(f_{1}/f_{0}+1\right)\\ d_{2}=\left(\phi_{2}^{2}-1\right)=\left(f_{2}/f_{0}-1\right)\left(f_{2}/f_{0}+1\right) \end{array}$$

Let

$$\Delta f_{1}=f_{0}-f_{1};\;\Delta f_{2}=f_{2}-f_{0}.$$

Then the limiting frequency increment is given by:

$$\Delta f=\Delta f_{1}+\Delta f_{2}.$$

The asymmetry between the limits *f*_1_ and *f*_2_ relative to *f*_0_ may be defined as:

$$A_{s}=\frac{\Delta f_{1}+\Delta f_{2}}{2f_{0}}.$$

From these definitions:

$$\frac{\Delta f}{f_{0}}=\frac{d_{2}-d_{1}}{2\left(1-A_{s}\right)}.$$

The solution for the quantity *(ϕ*^2^*—*1) yields, as the roots:

$$\begin{array}{l} d_{1}=\frac{-1}{2Q_{0}^{2}}-\frac{1}{Q_{0}}\sqrt{\left(e^{\alpha }-1\right)+1/4Q_{0}^{2}}\\ d_{2}=\frac{-1}{2Q_{0}^{2}}+\frac{1}{Q_{0}}\sqrt{\left(e^{\alpha }-1\right)+1/4Q_{0}^{2}} \end{array}$$

By applying the definitions for Δ*f*_1_, Δ*f*_2_ and *A_s_* to the roots *d*_1_ and *d*_2_, the value of *A_s_* is found to be

$$A_{s}=1-\sqrt{1-\frac{1}{4}{\left(\frac{\Delta f}{f_{0}}\right)}^{2}-\frac{1}{2Q_{0}^{2}}}.$$

No approximation has been used to this point. However, the second and third terms under the radical are much smaller than the first. When they are removed from the radical, by approximation:

$$A_{s}\approx \frac{1}{8}{\left(\frac{\Delta f}{f_{0}}\right)}^{2}+\frac{1}{4Q_{0}^{2}}$$

Now, in the expression for Δ*f*/*f*_0_ the term (1—*A_s_*) appears in the denominator. *A_s_* is composed of terms that are quite small relative to unity, so that a series approximation of one term is sufficient, and we may place it in the numerator, obtaining:

$$\frac{\Delta f}{f_{0}}=\frac{1}{Q_{0}}\sqrt{e^{\alpha }-1+\frac{1}{4Q_{0}^{2}}}\left(1+A_{s}\right).$$

Inspection of this expression shows that a further simplifying approximation may be made in *A_s_*, for it is clear that the term *(e^α^—* 1) is also rather small. Clearly the term in *A_s_* that contains the square of Δ*f*/*f*_0_ will in general be less than half as large as 1/4*Q*_0_^2^. Thus, the major term in *A_s_* is just the quantity 1/4*Q*_0_^2^. We have discarded this quantity from the equations shown in the text.

In order to find the limitations that bound the application of the equations given in the text, we must find the conditions under which the term 1/4*Q*_0_^2^ is indeed small.

When *Q*_0_ is taken as large as permitted by the least time interval in which an observation may take place, the relation between the resolution limit along the time axis and the energy resolution limit is

$$\frac{\Delta t}{T_{0}}=\frac{\alpha Q_{0}}{2\pi }$$

and, thus, in the limit:

$$\frac{1}{4Q_{0}^{2}}=\frac{\alpha^{2}T_{0}^{2}}{16\pi^{2}\Delta t^{2}}.$$

The approximation for Δ*f*/*f*_0_ will be poorest for the condition under which the discarded term is largest re (*e^α^—*1). From this limiting condition, we can find the bounds imposed upon the least time interval for which these derivations are applicable. Let

$$\frac{\alpha^{2}T_{0}^{2}}{16\pi^{2}\Delta t^{2}}÷\left(e^{\alpha }-1\right)=M\frac{\alpha^{2}}{\left(e^{\alpha }-1\right)}$$

where *M* represents the terms that do not contain *α*. Differentiating the right-hand term with respect to *α* and setting it equal to zero, one finds two solutions, the trivial one of *α* = 0 and the equation

$$\left(\alpha -2\right)\;e^{\alpha }+2=0.$$

A graphical solution gives as the most unfavorable condition, *α* =1.6.

This solution represents a condition in which Δ*E*/*E*_0_ would be very large; in fact, where noise is the limitation, the condition implies a signal-to-noise ratio of approximately 0.2. For this limit:

$$\left(e^{\alpha }-1\right)=e^{1.6}-1=4.$$

Thus, for a precision limit of about 12½ percent, we require only that the product α^2^ *M*< 1, or:

$$2.56\cdot {\left(\frac{T_{0}}{\Delta T}\right)}^{2}\cdot \frac{1}{16\pi^{2}}<1.$$

For times of observation comparable to one full period and even less, and in almost any condition in which *Q*_0_> 1, the term under the radical sign that we have discarded is in fact negligible.
